# Real-world outcomes of oral anticoagulation in patients with atrial fibrillation at high risk of both bleeding and stroke: observational evidence from three international registries from middle East, Europe and Asia-Pacific

**DOI:** 10.1007/s11239-025-03228-6

**Published:** 2025-12-22

**Authors:** Amir Askarinejad, Tommaso Bucci, Enrico Tartaglia, Steven H. M. Lam, Michele Rossi, Manlin Zhao, Hung-Fat Tse, Majid Haghjoo, Giuseppe Boriani, Tze-Fan Chao, Gregory Y. H. Lip

**Affiliations:** 1 Liverpool Heart & Chest Hospital, Liverpool Centre for Cardiovascular Sciences at University of Liverpool, Liverpool John Moores University, Liverpool, UK; 2 Department of Clinical Internal, Anesthesiologic and Cardiovascular Sciences, Sapienza University of Rome, Rome, Italy; 3 Division, Department of Biomedical, Metabolic and Neural Sciences, University of Modena and Reggio Emilia, Policlinico di Modena, Modena, Italy; 4 Department of Life, Health and Environmental Sciences, University of L’Aquila, L’Aquila, Italy; 5 Internal Medicine and Nephrology Division, ASL1 Avezzano-Sulmona-L’Aquila, San Salvatore Hospital, L’Aquila, Italy; 6 Department of Cardiology, Beijing Anzhen Hospital, Engineering Research Center of Medical Devices for Cardiovascular Diseases, Ministry of Education, National Clinical Research Center for Cardiovascular Diseases, Capital Medical University, Beijing, People’s Republic of China; 7 The University of Hong Kong, Hong Kong Special Administrative Region of China, Pok Fu Lam, Hong Kong; 8 Cardiac Electrophysiology Research Center, Rajaie Cardiovascular Institute, Tehran, Iran; 9 Cardiology Division, Department of Biomedical, Metabolic and Neural Sciences, Italy University of Modena and Reggio Emilia, Policlinico di Modena, Modena, Italy; 10 Division of Cardiology, Department of Medicine, Taipei Veterans General Hospital, Taipei, Taiwan; 11 Institute of Clinical Medicine and Cardiovascular Research Center, National Yang Ming Chiao Tung University, Aalborg, Taiwan; 12 Department of Clinical Medicine, Aalborg University, Taipei, Denmark; 13 Medical University of Bialystok, Bialystok, Poland

**Keywords:** Atrial fibrillation, Oral anticoagulants, Stroke risk, Bleeding risk

## Abstract

To evaluate the clinical course of patients with AF at high risk for both bleeding and stroke, according to OAC use. Data were analysed from three registries across the Middle East, Europe, and Asia-Pacific regions. The study only included ‘high risk’ patients with AF and CHA₂DS₂-VASc scores ≥ 2 and HAS-BLED scores ≥ 3, who were divided into two groups based on OAC use: OAC *users* and OAC *non-users*. Of the 2,535 patients (41.7% female; mean age 75.4 ± 7.8 years), 80.3% (*n* = 2,037) received OAC therapy. OAC *non‑users* showed significantly higher crude 1‑year event rates of all‑cause death (116 [23.3%]), MACE (96 [19.3%]) and major bleeding (31 [6.2%]); after multivariable adjustment, they had higher odds of all‑cause death (adjusted odds ratio (aOR) 2.23, 95% CI 1.65–3.01), MACE (aOR 1.92, 95% CI 1.38–2.64) and major bleeding (aOR 2.38, 95% CI 1.42–3.92) compared to OAC *users*. Enrolment in a non-European setting was associated with a lower risk of all-cause death (aOR 0.61, 95%CI 0.44–0.85) and MACE (aOR 0.42, 95%CI 0.28–0.62). In patients with AF at high risk of both bleeding and stroke, OAC *non-use* was associated with higher risk of adverse events and bleeding. Decisions on discontinuation of OACs in this subset of patients with AF should be cautiously made and such patients require careful re-evaluation and follow-up.

**Graphical abstract:**

**Figure:**
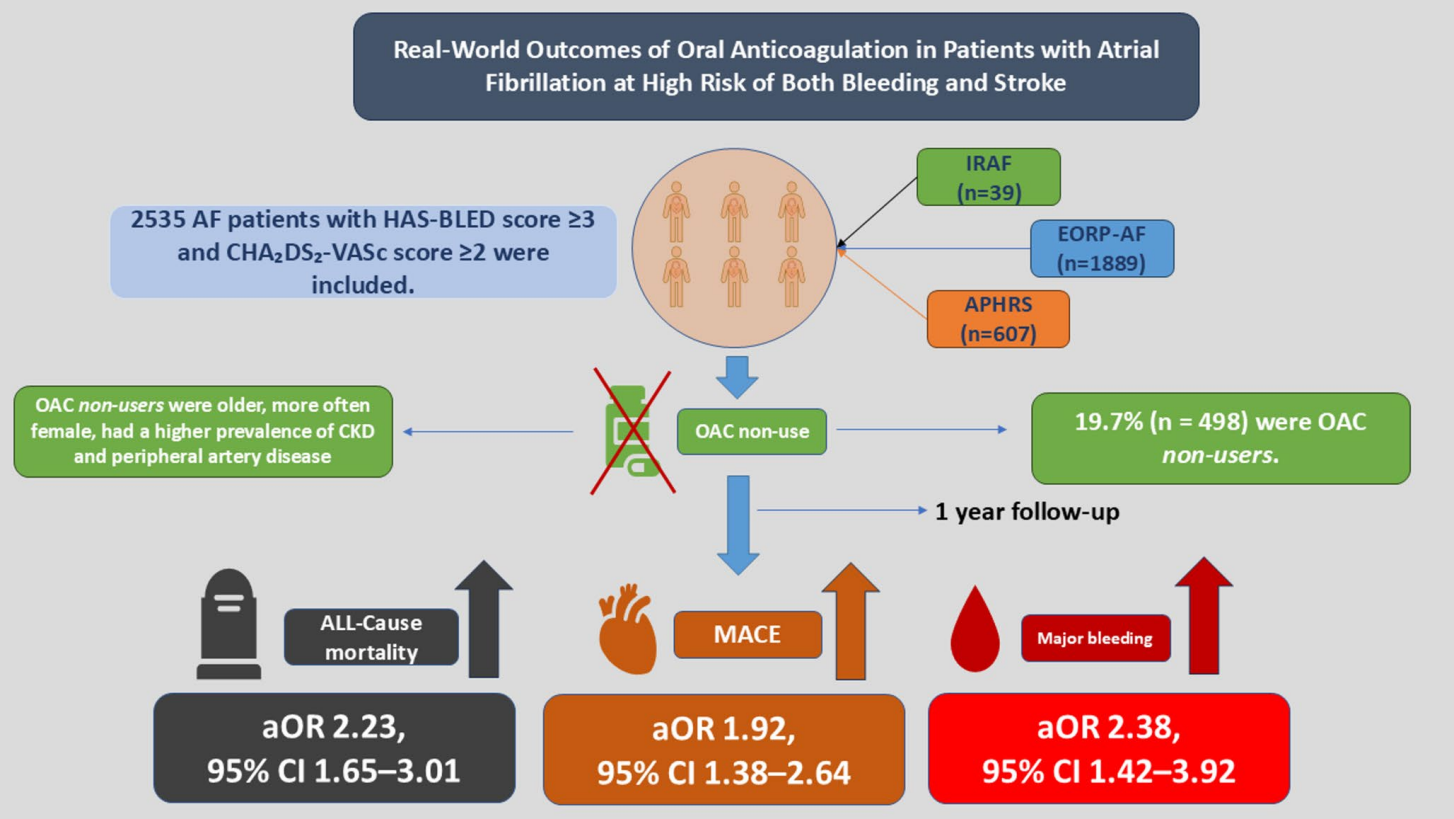
The study only included ‘high risk’ patients with AF and CHA₂DS₂-VASc scores ≥2 and HAS-BLED scores ≥3, who were divided into two groups based on OAC use: OAC users and OAC non-users. Of the 2,535 patients (41.7% female; mean age 75.4 ± 7.8 years), 80.3% (n=2,037) received OAC therapy. OAC non‑users showed significantly higher crude 1‑year event rates of all‑cause death (116 [23.3%]), MACE (96 [19.3%]) and major bleeding (31 [6.2%]); after multivariable adjustment, they had higher odds of all‑cause death (adjusted odds ratio [aOR] 2.23, 95% CI 1.65–3.01), MACE (aOR 1.92, 95% CI 1.38–2.64) and major bleeding (aOR 2.38, 95% CI 1.42–3.92) compared to OAC users

**Supplementary Information:**

The online version contains supplementary material available at 10.1007/s11239-025-03228-6.

## Introduction

Atrial fibrillation (AF) is the most common cardiac arrhythmia, with both its incidence and prevalence continuing to rise worldwide [1]. AF is associated with an increased risk of stroke and systemic thromboembolism, primarily due to blood stasis in the left atrium, which is caused by uncoordinated atrial contractions during AF episodes [2].

Oral anticoagulants (OACs) are the cornerstone of thromboembolic event prevention in patients with AF; however, their use must be carefully balanced against the risk of bleeding [3]. Indeed, in certain conditions characterised by the coexistence of multiple factors that increase bleeding risk, the net clinical benefit of OACs may be difficult to assess, potentially rendering their antithrombotic benefit uncertain.

Data on clinical outcomes in patients with AF at high risk of both stroke and bleeding remain limited, particularly regarding the potential impact of OAC use versus non-use. Moreover, some differences in outcomes are evident when comparing Asian and non-Asian cohorts of patients with AF [4–6], sometimes described as the ‘East Asian paradox’ in relation to antithrombotic therapy [7].

For these reasons, in this prospective cohort study, which analyses data from three registries across East Asia, the Middle East, and Europe, we aimed to evaluate the one-year risk of adverse events in patients at high risk of both thrombosis and bleeding. Our focus was not only on the clinical phenotype of these patients and the use of OAC at baseline but also on assessing geographical differences based on the enrolment settings.

## Methods

### Study population and registries

We included patients with AF from three large, prospective observational registries in Europe, East Asia, and the Middle East. The study design, baseline characteristics, and primary findings of these registries have been previously described in published reports [8–10]. These studies enrolled adult patients (aged ≥ 18) with documented AF on standard electrocardiograms within the 12 months prior to enrolment (Supplementary Table 1). Patients without 1-year follow-up data, with CHA₂DS₂-VASc score < 2 and HAS-BLED score < 3 among three registries were excluded.

### Thrombotic and haemorrhagic risk scores

The definition of thrombotic and haemorrhagic risk was based on the ESC and APHRS guidelines for AF management used at the time of enrolment (2015–2017), utilising the CHA₂DS₂-VASc and HAS-BLED risk scores [11]. Patients at high risk of thrombosis were defined as those with a CHA₂DS₂-VASc score ≥ 2, while patients at high risk of bleeding were defined as those with a HAS-BLED score ≥ 3.

### Cohort

Patients at high risk of thrombosis and bleeding were then categorised into two groups based on OAC use after enrolment: (i) those receiving OAC, and (ii) those not receiving OAC. Only patients with complete data on OAC use and full follow-up information were included.

### Outcomes

The primary outcome was net adverse clinical events (NACE), defined as a composite of all-cause death, a thromboembolic event or an acute coronary syndrome, or major bleeding. The secondary outcomes were the one-year risk of all-cause death, MACE, major bleeding, thromboembolic events, and acute coronary syndrome.

Major adverse cardiovascular event (MACE) was defined as the occurrence of either a thromboembolic event or an acute coronary syndrome.

### Statistical analysis

Categorical variables were reported as counts (percentages), while continuous variables were reported as mean ± standard deviation (SD). The Chi-square test was used to assess the significant differences in categorical variables. The independent-samples t-test was used to assess the significant difference of continuous data.

To assess potential multicollinearity among covariates, we calculated variance inflation factors (VIFs) for all predictors included in each multivariable logistic regression model. All VIFs were close to 1 (range 1.01–1.31), well below conventional thresholds (e.g. VIF > 5), indicating no relevant multicollinearity (Supplementary Table 2).

Univariate and multivariable logistic regression was used to: (i) investigate factors associated with OAC *non-use* and (ii) to assess the risk of adverse events in OAC *non-users* vs. OAC *users*. The multivariable model assessing OAC use was adjusted for age ≥ 75 years, female sex, underweight, hypertension, diabetes mellitus (DM), chronic kidney disease (CKD), peripheral artery disease, dyslipidaemia, heart failure, chronic obstructive pulmonary disease (COPD) prior thromboembolic events, history of major bleeding, antiplatelet therapy, and enrolment setting (APHRS and IRAF vs. EORP). A separate multivariable model assessing the risk of adverse events was adjusted for these same covariates, plus baseline OAC use. Variables were selected according to the components of the CHA_2_DS_2_-VASc score, established clinical risk factors for adverse events in AF, and anti-thrombotic treatments including OACs and anti-platelet drugs.

CKD was estimated using MDRD (Modification of Diet in Renal Disease Study Equation) formula and defined as a glomerular filtration rate (GFR) < 60 mL/min/1.73 m^2^. Subgroup analyses were performed to assess the association between OAC *non-use* in clinically relevant categories (based on the variables included in the multivariable logistic regression model) and the risk of all-cause death, MACE, and major bleeding. Moreover, to formally compare the adjusted odds of adverse outcomes between OAC *non-users* and each anticoagulant class (VKAs and non-vitamin K oral anticoagulants (NOACs)), we treated treatment as a three-level factor (“VKA”, “NOAC”, “No OAC”) in our multivariable logistic model. Subsequently, we used a Z-test to assess whether those two logs-ORs differed significantly from one another. VKAs and NOACs are both considered subcategories of OAC. Another subgroup analysis was performed on patients with high thromboembolic risk (CHA₂DS₂-VASc score of 3–5),very high thromboembolic risk (CHA₂DS₂-VASc score of 6–9), high bleeding risk (HAS-BLED score of 3–4), and very high bleeding risk (HAS-BLED score of 5–9) to assess the association of OAC non-use with composite outcome.

We performed a sensitivity analysis after excluding the patients from IRAF registry. Additional time-to-event sensitivity analyses were performed using Cox proportional hazards models to assess the robustness of the associations between baseline OAC use and adverse outcomes. For these analyses, we derived time-to-first-event endpoints over 1 year. For the composite outcome, follow-up time was defined from baseline to the earliest of thromboembolic event, acute coronary syndrome, major bleeding, or death, and censored at 365 days. For thromboembolic events, follow-up was censored at the first occurrence of major bleeding or death, or at 365 days, and only thromboembolic events occurring before these competing events contributed to the endpoint. Conversely, for major bleeding, follow-up was censored at the first thromboembolic event, acute coronary syndrome, or death, or at 365 days, and only bleeding events occurring prior to these events were counted. All Cox models were adjusted for the same covariates as the primary analyses.

Additionally, we applied inverse probability of treatment weighting (IPTW) to reduce confounding by indication when comparing OAC *users* with OAC *non-users*. A propensity score for OAC *use* was estimated for each patient using a logistic regression model using the same variables used in primary analysis. Stabilized IPTW weights were then calculated, and extreme weights were truncated at the 1 st and 99th percentiles to improve stability. These weights were used to construct a weighted “pseudo-population” in which baseline covariates were more comparable between treatment groups. Covariate balance before and after IPTW was evaluated using standardized mean differences (SMDs) (Supplementary Table 3, Supplementary Fig. 1).

To assess competing risks, we performed Fine–Gray subdistribution hazard models for 1-year thromboembolic events and major bleeding. For thromboembolic events, major bleeding and all-cause death were treated as competing events; for major bleeding, thromboembolic events, acute coronary syndrome, and all-cause death were treated as competing events. Time to event or competing event was truncated at 365 days; patients without an event by 1 year were censored. We estimated subdistribution hazard ratios (SHR) with 95% confidence intervals (CI), adjusting for same variables in primary analysis. Cumulative incidence functions (CIFs) for each outcome were plotted according to OAC status, and equality of CIFs between OAC use vs. non-use was assessed using Gray’s test.

All analyses were performed with R version 4.4.1 (R Core Team, Vienna, Austria). P-values < 0.05 were considered statistically significant.

## Results

**Fig. 1:**
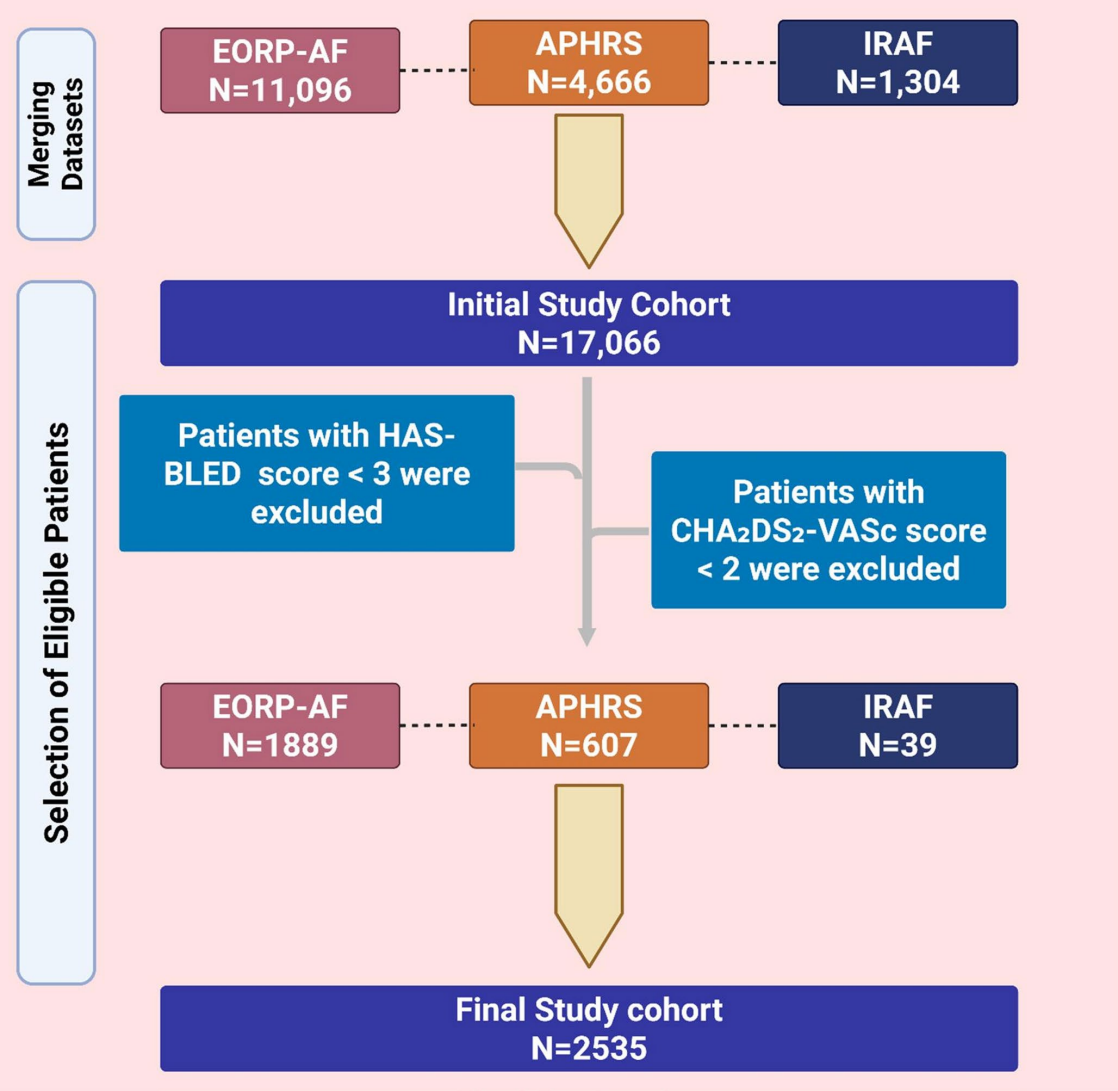
Flow chart of the study

After selecting eligible patients in three registries, 1889 patients from EORP-AF, 607 patients from APHRS, and 39 patients from IRAF were included (Fig. 1). A total of 2,535 patients (41.7% female; *n* = 1,057) with a mean age of 75.4 ± 7.8 years were analysed. The cohort included 1,889 (74.5%) European patients and 646 (25.5%) Asian patients, of whom 607 (23.9%) were East Asian and 39 (1.5%) were Middle Eastern (Supplementary Table 4).

Among the study population, 80.3% (*n* = 2,037) were OAC users, while 19.7% (*n* = 498) were OAC non-users. Compared to OAC users, OAC non-users were older, more often female, had a higher prevalence of CKD and peripheral artery disease, and were more frequently prescribed antiplatelet drugs (Supplementary Table 5).

### Factors associated with OAC use

In the multivariable logistic regression analysis, advanced age, female sex, prior bleeding, and enrolment in a non-European setting were associated with a lower likelihood of being prescribed OAC (Table 1). In contrast, dyslipidaemia and a history of thromboembolic events were associated with higher odds of OAC *use* (Table 1).

**Table 1:** Univariable and multivariable logistic regression for factors associated with the use of oral anticoagulants

	Univariable analysis OR (95% CI)	Multivariable analysis aOR* (95% CI)
Age ≥ 75 years	0.67 (0.55–0.82)	0.72 (0.57–0.91)
Female sex	0.81 (0.67–0.99)	0.73 (0.58–0.93)
Hypertension	0.90 (0.71–1.14)	0.89 (0.678–1.17)
Diabetes	0.92 (0.75–1.13)	1.05 (0.82–1.34)
CKD	0.82 (0.67–1.00.67.00)	0.90 (0.71–1.14)
PAD	0.69 (0.53–0.92)	0.77 (0.55–1.07)
Dyslipidaemia	1.07 (0.88–1.31)	1.43 (1.13–1.81)
COPD	1.24 (0.90–1.74)	1.33 (0.91–1.98)
Heart failure	0.83 (0.68–1.01)	1.05 (0.83–1.34)
Thromboembolic events	1.60 (1.27–2.04)	1.55 (1.18–2.05)
Haemorrhagic events	0.59 (0.47–0.75)	0.46 (0.35–0.61)
Antiplatelet use	0.14 (0.11–0.18)	0.13 (0.10–0.16)
Enrolment setting (APHRS + IRAF vs. EORP)	0.91 (0.73–1.14)	0.73 (0.56–0.96)

APHRS: Asian-Pacific Heart Rhythm Association, CKD: Chronic Kidney Disease, COPD: Chronic Obstructive Pulmonary Disease), IRAF: Iranian Registry of Atrial Fibrillation, EORP: EURObservational Research Programme, PAD: Peripheral Artery Disease. *: adjusted for age ≥ 75 years, female sex, hypertension, diabetes, chronic kidney disease, peripheral artery disease, dyslipidaemia, chronic obstructive pulmonary disease, heart failure, thromboembolic events, haemorrhagic events, antiplatelet use, and enrolment setting (APHRS + IRAF vs. EORP)

### Survival analysis

After one-year of follow-up, the following events occurred: 380 (15.0%) all-cause deaths, 316 (12.5%) MACE, 104 (4.1%) major bleeding, 84 (3.3%) thromboembolic events, and 136 (5.4%) acute coronary syndromes.

OAC *non-users* showed a higher one-year incidence of all primary and secondary outcomes compared to OAC *users* (Table 2). On univariable logistic regression, the higher risks of primary and secondary outcomes in OAC *non-users compared* to OAC *users* were confirmed, except for thromboembolic events that showed a non-statistically significant trend (Table 2).

**Table 2:** One-year incidence and comparative risk of clinical outcomes in oral anticoagulant (OAC) *users* versus OAC *non-users*

Outcome		One Year Incidence *N* (%)	*P*-value	Univariable Analysis OR (95% ci)	Multivariable Analysis OR (95% CI) *
NACE	OAC *users*	400 (19.6%)	< 0.001	Reference	Reference
	OAC *non-users*	167 (33.5%)		2.27 (1.81–2.84)	2.15 (1.63–2.82)
All-cause death	OAC *users*⸆	264 (13.0%)	< 0.001	Reference	Reference
	OAC *non-users*⸇	116 (23.3%)		2.19 (1.70–2.80)	2.23 (1.65–3.01)
MACE	OAC *users*	220 (10.8%)	< 0.001	Reference	Reference
	OAC *non-users*	96 (19.3%)		2.12 (1.62–2.76)	1.92 (1.38–2.64)
Major bleeding	OAC *users*	73 (3.6%)	< 0.001	Reference	Reference
	OAC *non-users*	31 (6.2%)		1.90 (1.21–2.9)	2.38 (1.42–3.92)
Thromboembolic events	OAC *users*	63 (3.1%)	0.152	Reference	Reference
	OAC *non-users*	21 (4.2%)		1.50 (0.88–2.44)	2.53 (1.34–4.61)
Acute coronary syndrome	OAC *users*	85 (4.2%)	< 0.001	Reference	Reference
	OAC *non-users*	51 (10.2%)		2.83 (1.96–4.06)	2.09 (1.34–3.25)

⸆:2037 patients were OAC *users*, ⸇:498 patients were OAC *non-users*. NACE: Net Adverse Clinical Events; MACE: Major adverse cardiovascular events; OR: odds ratio; CI: confidence interval; OAC: Oral anticoagulation. *: adjusted for age ≥ 75 years, female sex, hypertension, diabetes, chronic kidney disease, peripheral artery disease, dyslipidaemia, chronic obstructive pulmonary disease, heart failure, thromboembolic events, haemorrhagic events, antiplatelet use, OAC use, and enrolment setting (APHRS + IRAF vs. EORP)

On multivariable logistic regression analysis, compared to OAC *users*, OAC *non-users* showed higher risk of NACE, all-cause death, MACE, major bleeding, thromboembolic events, and acute coronary syndrome (Table 2).

### Geographical differences in the risk of adverse events

In multivariable logistic regression models for primary outcomes, enrolment in a non-European setting was associated with a lower risk of NACE, all-cause death, and MACE (Supplementary Table 6). The reduced likelihood of developing MACE in patients enrolled in a non-European setting was primarily driven by a lower risk of acute coronary syndrome rather than thromboembolic events (Supplementary Table 6).

### Subgroup analyses

**Fig. 2:**
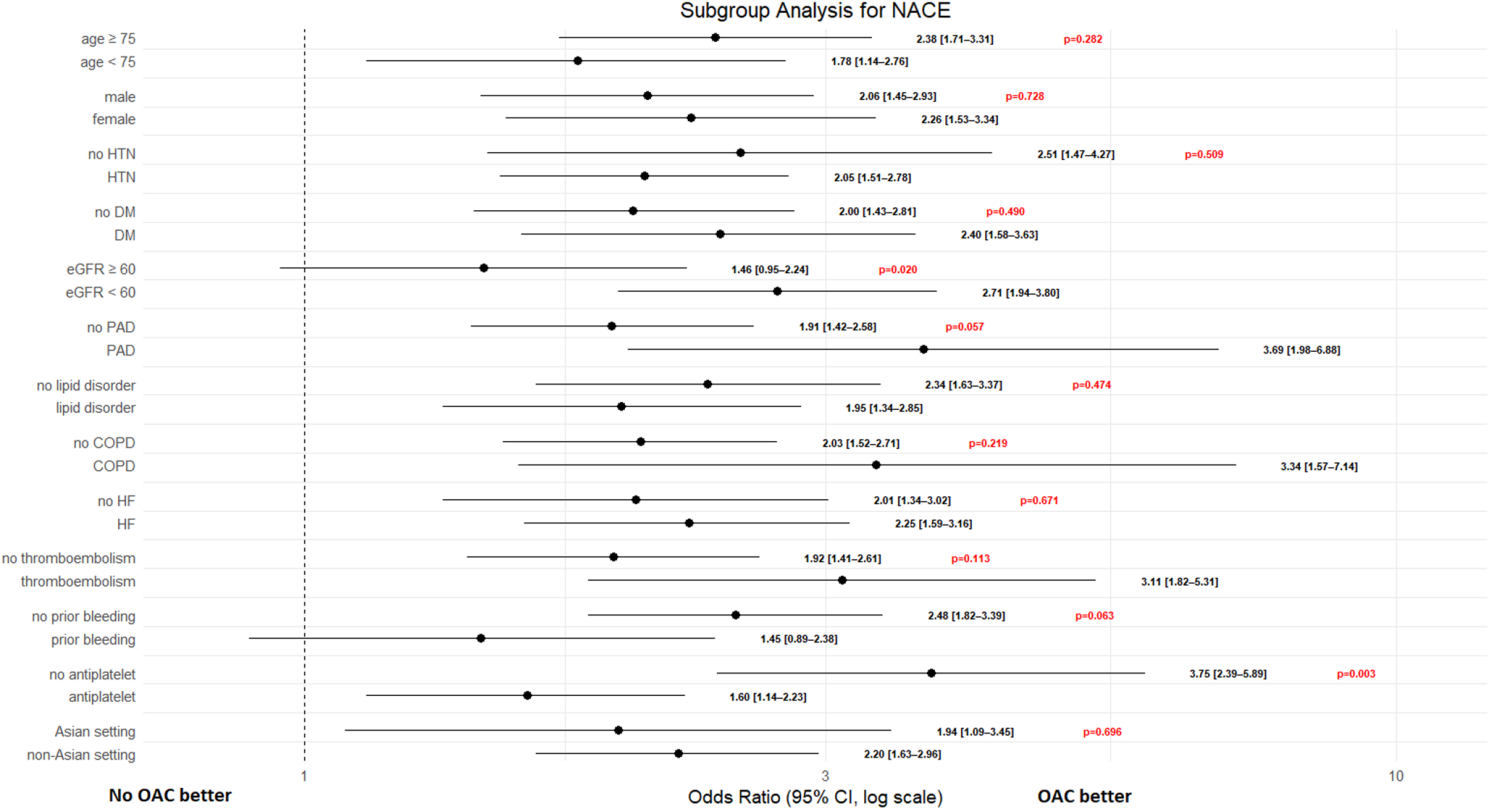
Subgroup analyses for the risk of NACE among OAC *users* versus OAC *non-users.* HTN: hypertension; COPD: chronic obstructive pulmonary disease; CKD: chronic kidney disease; HF: heart failure; PAD: peripheral artery disease

A significant interaction between OAC *use* and OAC *non-use* in patients without antiplatelet therapy and risk of NACE was observed (P for interaction < 0.05) (Fig. 2). When assessing the association between OAC *non-use* and the risk of all-cause death, we observed significant interaction (P for interaction < 0.05) between OAC use versus OAC non-use and each of the following factors: CKD status, COPD status, and antiplatelet prescription. Specifically, the association between OAC non-use and mortality was stronger in patients with CKD than in those without CKD, stronger in those with COPD than in those without COPD, and stronger in patients not prescribed antiplatelet drugs than in those who were. (Supplementary Fig. 2).

**Fig. 3:**
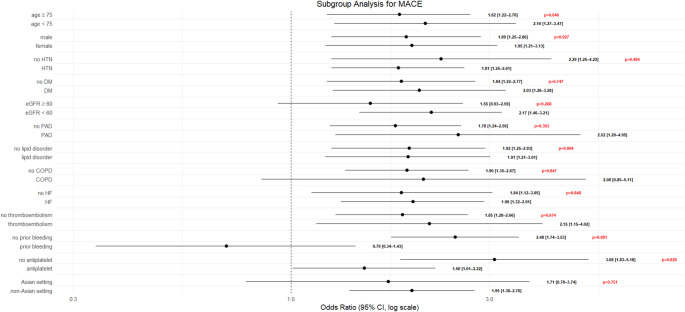
Subgroup analyses for the major adverse cardiovascular events (MACE) among OAC *users* versus OAC *non-users.* HTN: hypertension; COPD: chronic obstructive pulmonary disease; CKD: chronic kidney disease; HF: heart failure; PAD: peripheral artery disease

Regarding the risk of MACE, we observed significant interactions (P for interaction < 0.05) between OAC non-use and both prior bleeding status and antiplatelet prescription. Specifically, the association between OAC non-use and MACE was stronger in patients without prior bleeding events than in those with prior bleeding events, and stronger in those not prescribed antiplatelet drugs than in those who were. (Fig. 3).

**Fig. 4:**
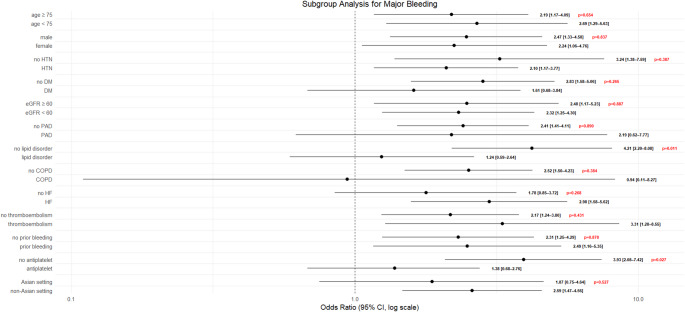
Subgroup analyses for the risk of major bleeding among OAC *users* versus OAC. *non-users.* HTN: hypertension; COPD: chronic obstructive pulmonary disease; CKD: chronic kidney disease; HF: heart failure; PAD: peripheral artery disease

Lastly, we observed significant interactions (P for interaction < 0.05) between OAC non-use and both dyslipidaemia status and antiplatelet prescription on the risk of major bleeding. Specifically, the association between OAC non-use and major bleeding was stronger in patients without dyslipidaemia than in those with dyslipidaemia, and stronger in those not prescribed antiplatelet drugs than in those who were. (Fig. 4).

Compared with VKA *users*, OAC *non-users* experienced higher risk of NACE, MACE, major bleeding, and acute coronary syndrome (Supplementary Table 7). Compared with NOAC *users*, OAC *non-users* had significantly higher risk of NACE, all-cause death, MACE, major bleeding, thromboembolic events, and acute coronary syndrome (Supplementary Table 7).

Higher risk of composite outcome was observed in patients with high thromboembolic risk (CHA₂DS₂-VASc score of 3–5) (adjusted OR 2.03, 95% CI 1.43–2.86), very high thromboembolic risk (CHA₂DS₂-VASc score of 6–9) (adjusted OR 3.30, 95% CI 1.97–5.53), high bleeding risk High bleeding risk (HAS-BLED score of 3–4) (adjusted OR 2.22, 95% CI 1.65–2.98), and very high bleeding risk (HAS-BLED score of 5–9) (adjusted OR 5.62, 95% CI 1.60–19.78) (Supplementary Table 8).

### Sensitivity analyses

After excluding patients from IRAF registry from the analytical sample size, the results were consistent with the primary results of the study. In time-to-event sensitivity analyses restricted to first events, OAC *non-use* remained independently associated with an increased risk of the composite outcome (aHR,1.46 95% CI 1.09–1.96) (Supplementary Table 9). In IPTW-weighted sensitivity analyses, OAC *non-use* was associated with increased risk of NACE (OR 2.92, 95% CI 2.27–3.76) and other outcomes; these IPTW-weighted findings were directionally and quantitatively consistent with the primary multivariable models (Supplementary Table 10).

In Fine–Gray models for 1-year thromboembolic events, with major bleeding or death as competing events, OAC *non-use* was not associated with higher thromboembolic risk compared with OAC *use* (SHR 1.18, 95% CI 0.47–2.94; *p* = 0.72). Enrolment in Asian setting was associated with a lower thromboembolic (SHR 0.26, 95% CI 0.08–0.83) (Supplementary Table 11). The cumulative incidence functions did not differ by OAC status (Gray test *p* = 0.97) (supplementary Fig. 3).

For 1-year major bleeding, using a Fine–Gray model with thromboembolism, acute coronary syndrome, and death as competing events, prior haemorrhagic events markedly increased bleeding risk (SHR 2.61, 95% CI 1.53–4.45; *p* < 0.001) (Supplementary Table 11). The cumulative incidence of major bleeding was numerically higher without OAC, but the difference was not statistically significant (Gray test *p* = 0.10) (supplementary Fig. 4).

## Discussion

The principal findings of our study are as follows: (i) patients with AF at high risk of both thrombosis and haemorrhagic events who were not prescribed with OACs exhibit a specific clinical phenotype characterised by advanced age, female predominance, and high prevalence of CKD, PAD, and antiplatelet use; ii) the factors independently associated with OAC *non-use* were advanced age, female sex, prior bleeding, and use of antiplatelets, whereas factors associated with higher likelihood of OAC *use* included prior thromboembolic events and dyslipidaemia; iii) after adjustment for confounders, OAC *non-users* were associated with a higher risk of all-cause death, MACE, major bleeding, thromboembolic events, acute coronary syndromes, and NACE compared to OAC *users*; iv) enrolment in non-European setting was significantly associated with a reduced risk of all-cause death, MACE, and acute coronary syndrome; v) The association of OAC non-use on the risks of all-cause death, MACE and major bleeding was greater in those not prescribed antiplatelet drugs compared to those prescribed antiplatelet drugs, with the highest risk of all-cause death in those with CKD and COPD.

The clinical phenotype observed in OAC *non-users* found in this study confirmed previous findings. For example, a post-hoc analysis of GARFIELD-AF registry evaluating the OAC use among different age groups (< 65, 65–74, 75–84, and ≥ 85 years) indicated that OACs were under prescribed in patients aged ≥ 85 years compared to those aged 65–84 years old [12]. The reasons behind this discrepancy are complex and involve several factors including multimorbidity, polypharmacy, and greater frailty, all of which make OAC management more challenging [13]. Future studies should incorporate formal frailty assessments to more accurately characterize the risk–benefit profile of OAC therapy in this double-risk AF population.

The lower use of OAC in females has also already been described [14]. Indeed, female sex has been associated not only with a higher thromboembolic risk in AF but also with under-treatment with OAC, which contributed to its inclusion in the original CHA₂DS₂-VASc model. Recent evidence suggests that female sex functions more as a risk modifier than as an independent risk factor [15–17]. Nielsen et al. demonstrated that the 5-year thromboembolic risk in women was comparable to men at a CHA₂DS₂-VA score of 0, but significantly higher at all scores ≥ 1, supporting the concept that female sex amplifies thromboembolic risk in the presence of additional non–sex-related stroke risk factors [16]. Although a non-sex-specific CHA₂DS₂-VA score has been proposed by the ESC guidelines —partly because including sex as a criterion complicates clinical decision-making and excludes non-binary, transgender, or hormone therapy patients— the potential for sex disparities in AF management suggest that we should remain cautious when assessing the risk of adverse events in high-risk populations, such as those patients with AF at high risk of both thrombosis and haemorrhage [18].

In our study, CKD was significantly more prevalent among OAC *non-users* and emerged as an independent predictor of not prescribing OACs. This is in accordance with previous studies. In a cohort of 6,544 patients with new-onset AF and CKD, followed for a median of 267 days, only 22.5% were prescribed OACs [19]. A post-hoc analysis of the ORBIT-AF registry showed that patients with stage IV or V CKD had a higher prevalence of OAC non-use than those without or with mild (stage III) CKD [20]. Furthermore, in patients with AF on dialysis, the randomised AXADIA–AFNET 8 trial [21] and the meta-analysis by Zimmerman et al. [22] have shown that any potential reduction in thromboembolic events with oral anticoagulation may be offset by a markedly increased bleeding risk, resulting in an uncertain net clinical benefit in this population. A recent meta-analysis of 4 randomized controlled trials on 486 dialysis-dependent patients with AF showed that factor Xa inhibitors were associated with a lower risk of intracranial bleeding compared with vitamin K antagonists [23].

This can be explained by the pro-haemorrhagic state described in CKD patients, due to the high prevalence of vascular diseases —often related with combined antithrombotic regimens with antiplatelets agents —impaired platelet function (widely reported, especially in end-stage renal disease) [24], and the continued preference for vitamin K antagonists, which often remain the treatment of choice in more advanced CKD stages despite evidence supporting the efficacy and safety of NOACs in patients with creatinine clearance as low as 25 ml/min [25, 26].

Taken together, our results regarding the clinical phenotype of OAC *non-users*, were further corroborated by a cluster analysis of 8,962 patients with AF not receiving anticoagulation, which showed as the cluster with the highest CHA_2_DS_2_-VASc and HAS-BLED scores had a female majority (52.7%), advanced age (77.19 ± 10.3), significantly lower eGFR [27].

A robust body of evidence has demonstrated a strong association between CKD and vascular disorders such as PAD [28]. Moreover, it has been shown that the presence of PAD in CKD patients is associated with a high use of antiplatelet therapy, which partially explaining why this treatment was independently related to the under-prescription of OACs in our population [29–31]. This may be explained by clinicians selecting antiplatelet therapy instead of OACs in patients with AF at high risk of bleeding, despite a lack of support from current literature and guidelines. Conversely, in our study, lipid disorders and prior thromboembolism were independently associated with higher OAC use. This may be explained by the pro-atherogenic effect of dyslipidaemia and the need for secondary thromboprophylaxis in patients who have experienced a prior stroke or systemic embolism [32, 33].

Although OAC therapy remains the cornerstone of stroke prevention in patients with AF [34], evidence regarding the safety and efficacy of OACs in patients with AF at higher risk of bleeding is limited [35, 36]. In line with our findings that OAC *non‑use* was associated with higher one‑year risks of all‑cause death, MACE and major bleeding, a prospective cohort study enrolling 4,777 patients with a HAS-BLED score ≥ 3, found that the 1,062 (22.2%) patients who discontinued OACs were at higher risk of ischaemic stroke, major bleeding, and all-cause death, compared to those who continued OACs [37]. A retrospective study on more than 29,000 patients with high clinical complexity —defined as the coexistence of advanced age, high frailty, CKD or prior haemorrhages —showed similar results, with higher risks of all-cause death and MACE, and a comparable risk of bleeding in patients who discontinued OACs compared to those who continued OAC [36]. Moreover, the beneficial effect of OACs was also demonstrated in patients with AF with high bleeding risk (HAS-BLED score ≥ 3) but intermediate stroke risk (CHA₂DS₂-VASc score of 1 for males or 2 for females), were OAC use was still associated with a reduced risk of composite adverse events, including ischaemic stroke, intracranial haemorrhage and mortality [38].

Concerns about haemorrhagic events in patients at high-risk of both bleeding and ischaemic events may lead to under-prescription of OACs, highlighting how challenging it can be for physicians to make appropriate decisions regarding OAC therapy [39]. Thus, despite evidence supporting OAC therapy even in these high-risk patients, some clinicians may remain hesitant to prescribe OACs due to the elevated haemorrhagic risk. Noteworthy, ELDERCARE-AF trial demonstrated that very low-dose edoxaban 15 mg once daily in very elderly (≥ 80 years of age) patients with AF at high bleeding risk significantly reduced stroke or systemic embolism compared with placebo, with an acceptable increase in major bleeding, supporting tailored low-dose DOAC therapy as a potential alternative to complete withdrawal of OAC in such high-risk patients [40].

Non-pharmacological options such as left atrial appendage occlusion (LAAO), have been considered as an alternative in those who are at high risk of stroke who have contraindications to OAC therapy. Among the trials assessing LAAO versus OACs, PRAGUE-17 trial had the most similar study population to our study (CHA₂DS₂-VASc score ≥ 3, HAS-BLED score ≥ 2) concluded non-inferiority of LAAO versus NOACs in patients with relative contraindications (e.g., frailty, history of bleeding) and those reluctant or intolerant to long-term OAC [41].

Our findings regarding significantly reduced risk of all-cause death, MACE, and acute coronary syndrome in patients who were enrolled in an non-European setting is supported by a post-hoc analysis from the GARFIELD-AF registry [42], that demonstrated as patients with AF enrolled in Asian centres had lower rates of all-cause mortality compared to those enrolled in other regions. Notably, OACs were more effective in reducing mortality and stroke among Asian patients, suggesting potential ethnic or regional variations in treatment outcomes and responses [42]. Our results confirmed also the results of another post-hoc analysis from the GLORIA-AF registry that demonstrated as compared to Western patients, Asian patients were associated with significantly lower risk of the primary composite outcome of all-cause death and MACE [43]. Racial disparities in AF management, suboptimal anticoagulation in Asian patients, and differing baseline health profiles have all contributed to the evolving concept of the “East Asian Paradox” [42]. Therefore, considering our findings alongside the aforementioned studies—and the growing evidence of ethnic-specific genetic and healthcare system factors—region-specific risk–benefit assessments are needed to guide personalized anticoagulation strategies in patients with AF at high risk for both bleeding and stroke.

One of the noteworthy findings of our study was the greater detrimental effect of OAC *non-use* on the risk of all-cause death in patients with CKD and COPD compared to those without these comorbidities. This may be explained by the higher baseline thromboembolic risk, heightened systemic inflammation, greater burden of cardiovascular comorbidities, and shared pathophysiological mechanisms, which together exacerbates harm in patients with CKD or COPD who are not anticoagulated [44, 45]. Another finding from the subgroup analysis was a more substantial effect of OAC *non-use* on bleeding in patients without dyslipidaemia compared to those with dyslipidaemia. This may reflect that patients with dyslipidaemia are more likely to receive statin therapy, which has been shown to confer a protective effect against bleeding in patients with AF [46, 47].

A more pronounced effect of OAC *non-use* on the risk of all-cause death, MACE, and major bleeding was found in patients who were not prescribed with antiplatelet drugs compared to those who were. Current guidelines and existing literature recommend OACs as the first-line therapy for stroke prevention in patients with AF and advise against using antiplatelet therapy as an alternative antithrombotic option [48]. Notably, in patients with AF, antiplatelet therapy has been shown to carry a higher risk of stroke compared to OACs, yet it has been associated with a lower risk of stroke compared to placebo, albeit with a higher risk of bleeding [49, 50]. Another possible explanation for the higher risk of adverse events of associated with OAC *non-use* in patients not receiving antiplatelet therapy could be a high baseline comorbidity burden and greater frailty, leading to an elevated untreated thromboembolic risk, and consequently higher rates of all-cause death and MACE. Decisions on discontinuation of OACs in this subset of patients with AF should be cautiously made and such patients require careful re-evaluation and follow-up.

Our study highlights the clinical complexity associated with AF, should be managed through a comprehensive, holistic, and integrated approach such as the evidence-based ABC pathway (A: Avoid stroke with anticoagulation; B: Better symptom management; C: Cardiovascular and comorbidity optimisation), which is supported by randomised trial evidence [51, 52]. A tailored approach—selecting optimal anticoagulation, rhythm or rate control, and cardiovascular risk management while balancing benefits and harms—improves outcomes in patients with AF, even those with high clinical complexity.

### Limitations

The relatively short follow-up period may have limited our ability to fully assess the long-term association between different antithrombotic strategies and clinical outcomes. Our findings are subject to inherent limitations, including potential residual confounding and survival selection bias. Subgroup analyses are exploratory and should be interpreted with caution given the study’s observational design and potential residual confounding. Furthermore, excluding patients without complete follow-up and those who died before registry enrollment may have introduced immortal‑time and additional selection biases. Since OAC use was defined based on patients’ treatment status at enrollment, we did not account for discontinuation or initiation of OAC therapy during follow-up. We did not have longitudinal information on the timing and duration of triple or dual therapy, the specific P2Y12/ADP inhibitor used, or the duration of combination therapy with OAC/VKA. This limitation should be acknowledged, as dynamic changes in OAC use may have influenced our results. Events that occurred between AF diagnosis and registry enrollment were not captured, potentially leading to underestimation of true event rates. The higher rate of bleeding events among OAC non‑users likely reflects indication bias: clinicians tend to withhold anticoagulation in patients perceived to be at especially high bleeding risk, who might have had unmeasured characteristics—such as advanced frailty, fall risk, cognitive impairment, or other comorbidities not captured in our registries—that predispose them to bleeding independently of OAC use. Therefore, these findings should be interpreted cautiously, recognizing that residual confounding by these unrecorded factors may drive the observed association. It is important to note that the higher bleeding risk observed in OAC *non-users* represents an association rather than a causal effect, given the observational nature of our study.

## Conclusions

OAC non-use in patients with AF at high-risk of both bleeding and stroke is associated with higher risk of adverse outcomes, including all-cause death, MACE, and major bleeding. Decisions on discontinuation of OACs in this subset of patients with AF should be cautiously made and such patients require careful re-evaluation and follow-up.

## Supplementary Information

Below is the link to the electronic supplementary material.

Supplementary Material 1.

Supplementary Figure 1. Propensity score distributions before and after inverse probability of treatment weighting (IPTW).

Supplementary Material 3Supplementary Figure 2. Subgroup analyses for the risk of all-cause death among OAC users versus OAC non-users

Supplementary Figure 3. Cumulative incidence functions by OAC status for 1-year thromboembolic events with major bleeding or death as competing events.

Supplementary Figure 4. Cumulative incidence functions by OAC status for 1-year major bleeding and competing events (thromboembolism, acute coronary syndrome, other cardiovascular events, or death).

## Data Availability

Data used in this research project will be available upon a reasonable request from the corresponding authors.
