# Supplementary material for: Real-world outcomes of oral anticoagulation in patients with atrial fibrillation at high risk of both bleeding and stroke: observational evidence from three international registries from middle East, Europe and Asia-Pacific
Source: J Thromb Thrombolysis. 2025 Dec 22;59(3):574–86. doi: 10.1007/s11239-025-03228-6 (PMC13246897; doi:10.1007/s11239-025-03228-6)
Supplement: Supplementary file 1 — Supplementary Material 1. [file 11239_2025_3228_MOESM1_ESM.docx]

**Real-World Outcomes of Oral Anticoagulation in Patients with Atrial Fibrillation at High Risk of Both Bleeding and Stroke: Observational Evidence from Three International Registries from Middle East, Europe and Asia-Pacific**

Amir Askarinejad, Tommaso Bucci, Enrico Tartaglia, Steven H.M. Lam, Michele Rossi, Manlin Zhao, Hung-Fat Tse, Majid Haghjoo, Giuseppe Boriani, Tze-Fan Chao, Gregory Y. H. Lip

**Supplementary material**

**Supplementary Table 1. Details regarding the three atrial fibrillation registries of our study.**

| Registry |  |
| --- | --- |
| EURObservational Research Programme (EORP) AF General Long-Term Registry | In the European Society of Cardiology (ESC) and European Heart Rhythm Association (EHRA) EURObservational Research Programme (EORP) AF General Long-Term Registry, patients were enrolled from 250 centres across 27 countries between October 2013 and September 2016, with a pre-planned two-year follow-up(1). The registry aimed to comprehensively represent the European AF population by including diverse healthcare settings. Ethical approval was obtained for each participating country and site through the main institutions of the National Coordinators. The study adhered to the European Union Note for Guidance on Good Clinical Practice (CPMP/ECH/135/95) and the Declaration of Helsinki. |
| Asia-Pacific Heart Rhythm Society (APHRS) AF Registry | The Asia-Pacific Heart Rhythm Society (APHRS) AF Registry enrolled AF patients from 52 centres across five Southeast Asian countries. Patient recruitment began in late 2015 and continued until early 2017, with a one-year follow-up period(2). Ethical approval was granted by the respective local ethics committees in each participating country. |
| Iranian Registry of Atrial Fibrillation (IRAF) registry | The Iranian Registry of Atrial Fibrillation (IRAF) included consecutive AF patients who presented to outpatient clinics (including arrhythmia clinics and emergency departments) or were hospitalized for further evaluation and treatment at Rajaie Cardiovascular Medical and Research Institute(3). The registry gathered data from patients referred to a tertiary centre from all provinces of Iran, providing a representative sample of the Iranian AF population. Data collection was conducted through patient interviews and medical record reviews, with information entered into a web-based electronic database (<https://regitory.rhc.ac.ir>). |

At baseline, investigators collected data on demographics, comorbidities, and pharmacological treatments using a standardized electronic case report form (eCRF). The same eCRF, with harmonised variables, was used for data collection in both the EORP and APHRS registries. Data from the IRAF registry were integrated where methodological consistency allowed, and a merged dataset was created. However, in cases of discrepancies—such as variations in definitions, data collection methods, or other methodological differences—data were not merged to preserve the homogeneity and integrity of the study population. To ensure clarity throughout the manuscript, we refer to patients from the European registry as “EORP-AF,” those from the East Asian registry as “APHRS,” and those from the Middle Eastern registry as “IRAF.” This terminology reflects the source registry rather than the precise geographic origin of the patients. In all three registries, patients on VKA therapy were managed to a target INR of 2.0–3.0 for thromboembolism prevention; all other antithrombotic regimens were prescribed in accordance with the 2020 ESC atrial fibrillation guidelines. In EORP-APHRS major bleeding was defined based on occurrence of intracranial haemorrhage and major extracranial haemorrhage during follow-up. Major extracranial bleeding was defined as a bleeding event causing a drop in haemoglobin level >2 g/L, requiring blood transfusion or hospitalization occurring in any major organ system. In IRAF, Bleeding events were defined as the occurrence of any major bleeding (including gastrointestinal and intracranial bleeding), clinically relevant non-major bleeding (such as epistaxis), and minor bleeding (including bruising or superficial hematoma). A thromboembolic event was defined as the occurrence of stroke, transient ischaemic attack, or any peripheral embolism.

**Supplementary Table 2. Variance inflation factors (VIFs) for each predictor across multivariable logistic regression models.**

| **Predictor** | **NACE** | **All-cause death** | **MACE** | **Major bleeding** | **Thromboembolic events** | **Acute coronary syndrome** |
| --- | --- | --- | --- | --- | --- | --- |
| **OAC non-use** | 1.22 | 1.25 | 1.22 | 1.31 | 1.28 | 1.24 |
| **Antiplatelet therapy** | 1.21 | 1.24 | 1.2 | 1.29 | 1.26 | 1.21 |
| **Heart failure** | 1.08 | 1.09 | 1.08 | 1.12 | 1.1 | 1.09 |
| **Enrollment setting** | 1.08 | 1.09 | 1.06 | 1.1 | 1.09 | 1.08 |
| **Chronic kidney disease** | 1.07 | 1.07 | 1.07 | 1.09 | 1.07 | 1.08 |
| **Dyslipidaemia** | 1.07 | 1.07 | 1.07 | 1.07 | 1.07 | 1.08 |
| **Diabetes mellitus** | 1.06 | 1.06 | 1.06 | 1.06 | 1.05 | 1.06 |
| **Female sex** | 1.05 | 1.05 | 1.04 | 1.04 | 1.03 | 1.05 |
| **Peripheral arterial disease** | 1.05 | 1.06 | 1.06 | 1.06 | 1.04 | 1.06 |
| **Hypertension** | 1.04 | 1.04 | 1.04 | 1.04 | 1.04 | 1.04 |
| **History of major bleeding** | 1.04 | 1.04 | 1.04 | 1.06 | 1.03 | 1.04 |
| **Age ≥ 75 years** | 1.03 | 1.03 | 1.03 | 1.04 | 1.04 | 1.03 |
| **COPD** | 1.03 | 1.04 | 1.04 | 1.03 | 1.01 | 1.04 |
| **History of thromboembolism** | 1.03 | 1.03 | 1.03 | 1.03 | 1.03 | 1.03 |

**Supplementary Table 3. Baseline characteristics before and after inverse probability of treatment weighting (IPTW).**

|  | **Before IPTW** | | | **After IPTW** | | |
| --- | --- | --- | --- | --- | --- | --- |
|  | **OAC *use***  **(N = 2037)** | **OAC *non-use***  **(N = 498)** | **SMD** | **OAC *use***  **(Σw = 1745.18)** | **OAC *non-use***  **(Σw = 370.64)** | **SMD** |
| **Age (years)** | 75.02 ± 7.58 | 76.93 ± 8.57 | 0.236 | 75.21 ± 7.33 | 74.12 ± 9.57 | 0.128 |
| **Body mass index (kg/m²)** | 27.38 (4.93) | 26.46 (5.13) | 0.183 | 27.14 (4.82) | 26.95 (4.99) | 0.038 |
| **Female sex (%)** | 829 (40.7) | 228 (45.8) | 0.103 | 705.7 (40.4) | 159.5 (43.0) | 0.053 |
| **Hypertension (%)** | 1554 (76.7) | 386 (78.5) | 0.042 | 1338.7 (76.7) | 284.0 (76.6) | 0.002 |
| **Diabetes mellitus (%)** | 702 (34.8) | 181 (36.6) | 0.038 | 604.9 (34.7) | 130.3 (35.2) | 0.011 |
| **Chronic kidney disease (eGFR <60 mL/min) (%)** | 1066 (52.3) | 285 (57.2) | 0.099 | 922.9 (52.9) | 202.6 (54.7) | 0.036 |
| **Peripheral arterial disease (%)** | 235 (11.8) | 78 (16.2) | 0.127 | 229.5 (13.2) | 59.0 (15.9) | 0.078 |
| **Dyslipidaemia (%)** | 1038 (52.4) | 241 (50.6) | 0.036 | 903.9 (51.8) | 181.9 (49.1) | 0.055 |
| **COPD (%)** | 234 (11.5) | 47 (9.5) | 0.066 | 192.5 (11.0) | 38.5 (10.4) | 0.021 |
| **Heart failure (%)** | 1020 (50.5) | 272 (55.3) | 0.095 | 865.2 (49.6) | 177.3 (47.8) | 0.035 |
| **History of thromboembolism (%)** | 617 (30.5) | 105 (21.5) | 0.207 | 483.4 (27.7) | 84.8 (22.9) | 0.111 |
| **History of major bleeding (%)** | 367 (18.2) | 133 (27.3) | 0.218 | 351.7 (20.1) | 77.5 (20.9) | 0.019 |
| **Antiplatelet therapy (%)** | 608 (29.9) | 373 (74.9) | 1.010 | 659.5 (37.8) | 146.3 (39.5) | 0.034 |
| **Asian ethnicity (%)** | 512 (25.1) | 134 (26.9) | 0.040 | 440.6 (25.2) | 94.2 (25.4) | 0.004 |

“Before IPTW” columns show the crude (unweighted) distributions in OAC users and non-users with standardized mean differences (SMD). “After IPTW” columns show the weighted sums (Σw) for OAC and no OAC groups and the corresponding SMDs after IPTW using stabilized, truncated weights. An absolute SMD <0.10 was considered to indicate adequate balance between treatment groups. COPD: chronic obstructive pulmonary disease; eGFR: estimated glomerular filtration rate.

**Supplementary Table 4. Baseline characteristics of the study population.**

|  | | **Total study population (n=2535)** | **EORP-AF**  **(n=1889)** | **IRAF**  **(n=39)** | **APHRS**  **(n=607)** | **P-value** |
| --- | --- | --- | --- | --- | --- | --- |
| **Age, mean ± SD** | | 75.4 ± 7.8 | 75.3 ± 7.4 | 67.1 ± 10.7 | 76.1 ± 8.5 | <0.001 |
| **Female gender** | | 1057 (41.7%) | 791 (41.9%) | 20 (51.3%) | 246 (40.5%) | 0.399 |
| **BMI, mean ± SD** | | 27.2 ± 5.0 | 28.0 ± 4.8 | 25.4 ± 3.2 | 24.7 ±4.0 | <0.001 |
| **Blood pressure** | **Systolic** | 138.8 ± 24.1 | 139.7 ± 24.1 | 120.3 ± 32.0 | 137.2 ± 23.0 | <0.001 |
|  | **Diastolic** | 78.9 ± 13.8 | 80.6 ± 13.8 | 79.1 ± 8.2 | 73.7 ± 12.8 | <0.001 |
| **Symptoms** | **Palpitation** | 638 (25.2%) | 546 (28.9%) | 26 (66.7%) | 66 (10.9%) | <0.001 |
|  | **Syncope** | 54 (2.1%) | 47 (2.5%) | 0 (0.0%) | 7 (1.2%) | 0.091 |
|  | **Dyspnea** | 673 (26.5%) | 598 (31.7%) | 11 (28.2%) | 64 (10.5%) | <0.001 |
|  | **Chest pain** | 288 (11.4%) | 252 (13.3%) | 0 (0.0%) | 36 (5.9%) | <0.001 |
|  | **Dizziness** | 239 (9.4%) | 205 (10.9%) | 3 (7.7%) | 31 (5.1%) | <0.001 |
|  | **Fatigue** | 423 (16.7%) | 404 (21.4%) | 1 (2.6%) | 18 (3.0%) | <0.001 |
|  | **Anxiety** | 145 (5.7%) | 139 (7.4%) | 0 (0.0%) | 6 (1.0%) | <0.001 |
| **Comorbidities and risk factors** | **Hypertension** | 1940 (76.5%) | 1411 (74.7%) | 26 (66.7%) | 503 (82.9%) | <0.001 |
|  | **Diabetes** | 883 (34.8%) | 642 (34.0%) | 9 (23.1%) | 232 (38.2%) | 0.093 |
|  | **Dyslipidaemia** | 1279 (50.4%) | 942 (49.9%) | 8 (20.5%) | 329 (54.2%) | <0.001 |
|  | **Chronic kidney disease** | 1025 (40.4%) | 826 (43.7%) | 6 (15.4%) | 193 (31.8%) | <0.001 |
|  | **Heart failure** | 1292 (51.0%) | 1076 (57.0%) | 7 (17.9%) | 209 (34.4%) | <0.001 |
|  | **COPD** | 281 (11.1%) | 252 (13.3%) | 0 (0.0%) | 29 (4.8%) | <0.001 |
|  | **Peripheral artery disease** | 313 (12.3%) | 294 (15.6%) | 0 (0.0%) | 19 (3.1%) | <0.001 |
|  | **Smoking** | 165(6.5%) | 127(6.7%) | 2(5.1%) | 36(5.9%) | 0.327 |
| **CHA₂DS₂-VASc score** | **Mean ± SD** | 4.5 ± 1.5 | 4.5 ± 1.6 | 4.4 ± 1.4 | 4.3 ± 1.4 | 0.254 |
|  | **2** | 255 (10.1%) | 190(10.1%) | 4(10.3%) | 61(10.0%) |  |
|  | **3** | 474 (18.7%) | 339(17.9%) | 8(20.5%) | 127(20.9%) |  |
|  | **4** | 615 (24.3%) | 454(24.0%) | 7(17.9%) | 154(25.4%) |  |
|  | **5** | 562 (22.2%) | 430(22.8%) | 11(28.2%) | 121(19.9%) |  |
|  | **6** | 362 (14.3%) | 255(13.5%) | 6(15.4%) | 101(16.6%) |  |
|  | **7** | 195 (7.7%) | 160(8.5%) | 3(7.7%) | 32(5.3%) |  |
|  | **8** | 59 (2.3%) | 50(2.6%) | 0(0.0%) | 9(1.5%) |  |
|  | **9** | 13 (0.5%) | 11(0.6%) | 0(0.0%) | 2(0.3%) |  |
| **HAS-BLED Score** | **Mean ± SD** | 3.3 ± 0.6 | 3.3 ± 0.6 | 3.0 ± 0.3 | 3.3 ± 0.6 | 0.179 |
|  | **3** | 1886 (74.4%) | 1403 (74.3%) | 37 (94.9%) | 446 (73.5%) |  |
|  | **4** | 525 (20.7%) | 398 (21.1%) | 1 (2.6%) | 126 (20.8%) |  |
|  | **5** | 108 (4.3%) | 75 (4.0%) | 1 (2.6%) | 32 (5.3%) |  |
|  | **6** | 15 (0.6%) | 12 (0.6%) | 0 (0.0%) | 3 (0.5%) |  |
|  | **7** | 1 (0.04%) | 1 (0.1%) | 0 (0.0%) | 0 (0.0%) |  |
| **Echocardiography findings** | **LVEF, mean (SD)** | 51.7 ± 13.7 | 50.4 ± 13.8 | 43.6 ± 10.9 | 56.0 ± 12.3 | <0.001 |
|  | **LVEDD, mean (SD)** | 49.9 ± 10.6 | 51.9 ± 8.5 | 60.5 ± 11.1 | 47.9 ± 7.1 | <0.001 |
| **Antiplatelet therapy** | | 981 (38.7%) | 766 (40.6%) | 0 (0.0%) | 215 (35.4%) | <0.001 |
| **VKAs** | | 1367 (53.9%) | 1107 (58.6%) | 12 (30.8%) | 248 (40.9%) | <0.001 |
| **NOACs** | | 670 (26.4%) | 418 (22.1%) | 13 (33.3%) | 239 (39.4%) | <0.001 |
| **OACs** | | 2037 (80.3%) | 1525 (80.7%) | 25 (64.1%) | 487 (80.2%) | 0.035 |
| **ACEI** | | 1051(41.5%) | 910(48.2%) | 8(20.5%) | 133(21.9%) | <0.001 |
| **ARBs** | | 589(23.2%) | 399(21.1%) | 17(43.6%) | 173(28.5%) | <0.001 |
| **Beta-blockers** | | 1746(68.9%) | 1359(71.9%) | 28(71.8%) | 359(59.1%) | <0.001 |

Continuous variables are presented as mean (standard deviation), while categorical variables are presented as number (percentage).AF : Atrial Fibrillation; IRAF : Iranian Atrial Fibrillation Registry; EORP-AF : EURObservational Research Programme on Atrial Fibrillation; APHRS : Asia-Pacific Heart Rhythm Society Atrial Fibrillation Registry; BMI : Body Mass Index; COPD : Chronic Obstructive Pulmonary Disease; CHA₂DS₂-VASc : Congestive Heart Failure, Hypertension, Age ≥75 (2 points), Diabetes Mellitus, Stroke/TIA/Thromboembolism (2 points), Vascular Disease, Age 65-74, Sex Category (Female); HAS-BLED : Hypertension, Abnormal renal/liver function, Stroke, Bleeding, Labile INR, Elderly, Drugs/Alcohol; LVEF : Left Ventricular Ejection Fraction; LVEDD : Left Ventricular End-Diastolic Diameter; AS : Aortic Stenosis; AR : Aortic Regurgitation; MS : Mitral Stenosis; MR : Mitral Regurgitation; VKA : Vitamin K Antagonists; NOAC : Non-Vitamin K Oral Anticoagulants; ACEI : Angiotensin-Converting Enzyme Inhibitor; ARB : Angiotensin II Receptor Blocker.

**Supplementary Table 5. characteristics of the patients according to OAC therapy.**

|  | | **Total study population (n=2535)** | **OACs**  ***non-users* (n=498)** | **OAC *users* (n=2037)** | **P-value** |
| --- | --- | --- | --- | --- | --- |
| **Age** | | 75.4 ± 7.8 | 76.9 ± 8.6 | 75.0 ± 7.6 | <0.001 |
| **Female sex** | | 1057 (41.7%) | 228 (45.8%) | 829 (40.7%) | 0.044 |
| **BMI** | | 27.2 ± 5.0 | 26.4 ± 5.1 | 27.3 ± 4.9 | 0.457 |
| **Blood pressure** | **Systolic** | 138.8 ± 24.1 | 140.8 ± 24.6 | 138.3 ± 24.0 | 0.038 |
|  | **Diastolic** | 78.9 ± 13.7 | 78.5 ± 13.9 | 79.0 ± 13.7 | 0.49 |
| **Symptoms** | **Palpitation** | 638 (25.2%) | 131 (26.3%) | 507 (24.9%) | 0.552 |
|  | **Syncope** | 54 (2.1%) | 16 (3.2%) | 38 (1.9%) | 0.090 |
|  | **Dyspnoea** | 673 (26.5%) | 130 (26.1%) | 543 (26.7%) | 0.846 |
|  | **Chest pain** | 288 (11.4%) | 81 (16.3%) | 207 (10.2%) | <0.001 |
|  | **Dizziness** | 239 (9.4%) | 58 (11.6%) | 181 (8.9%) | 0.071 |
|  | **Fatigue** | 423 (16.7%) | 96 (19.3%) | 327 (16.1%) | 0.096 |
|  | **Anxiety** | 145 (5.7%) | 39 (7.8%) | 106 (5.2%) | 0.031 |
| **Comorbidities and risk factors** | **Hypertension** | 1940 (76.5%) | 386 (77.5%) | 1554 (76.3%) | 0.442 |
|  | **Diabetes** | 883 (34.8%) | 181 (36.3%) | 702 (34.5%) | 0.476 |
|  | **Dyslipidaemia** | 1279 (50.4%) | 241 (48.4%) | 1038 (51.0%) | 0.514 |
|  | **Chronic kidney disease** | 1025 (40.4%) | 221 (44.4%) | 804 (39.5%) | 0.048 |
|  | **Heart failure** | 1292 (51.0%) | 272 (54.6%) | 1020 (50.1%) | 0.066 |
|  | **COPD** | 281 (11.0%) | 47 (9.4%) | 234 (11.5%) | 0.233 |
|  | **Peripheral artery disease** | 313 (12.3%) | 78 (15.7%) | 235 (11.5%) | 0.011 |
|  | **Smoking** | 165 (6.5%) | 25 (5.0%) | 140 (6.9%) | 0.214 |
| **CHA₂DS₂-VASc score** | **Mean ± SD** | 4.5 ± 1.5 | 4.6 ± 1.5 | 4.4 ± 1.5 | 0.025 |
|  | **2** | 255 (10.1%) | 45 (9.0%) | 210 (10.3%) | 0.227 |
|  | **3** | 474 (18.7%) | 80 (16.1%) | 394 (19.3%) |  |
|  | **4** | 615 (24.3%) | 117 (23.5%) | 498 (24.4%) |  |
|  | **5** | 562 (22.2%) | 121 (24.3%) | 441 (21.6%) |  |
|  | **6** | 362 (14.3%) | 72 (14.5%) | 290 (14.2%) |  |
|  | **7** | 195 (7.7%) | 49 (9.8%) | 146 (7.2%) |  |
|  | **8** | 59 (2.3%) | 10 (2.0%) | 49 (2.4%) |  |
|  | **9** | 13 (0.5%) | 4 (0.8%) | 9 (0.4%) |  |
| **HAS-BLED Score** | **Mean ± SD** | 3.3 ± 0.6 | 3.4 ± 0.6 | 3.3 ± 0.6 | 0.107 |
|  | **3** | 1886 (74.4%) | 352 (70.7%) | 1534 (75.3%) | 0.226 |
|  | **4** | 525 (20.7%) | 120 (24.1%) | 405 (19.9%) |  |
|  | **5** | 108 (4.3%) | 24 (4.8%) | 84 (4.1%) |  |
|  | **6** | 15 (0.6%) | 2 (0.4%) | 13 (0.6%) |  |
|  | **7** | 1 (0.03%) | 0 (0.0%) | 1 (0.05%) |  |
| **Echocardiography findings** | **LVEF** | 51.7 ± 13.7 | 51.0 ± 14.0 | 51.8 ± 13.6 | 0.284 |
|  | **LVEDD** | 49.9 ± 10.6 | 48.6 ± 12.0 | 50.2 ± 10.2 | 0.013 |
| **Antiplatelet therapy** | | 981 (38.7%) | 373 (74.9%) | 608 (29.9%) | <0.001 |
| **VKAs** | | 1367 (53.9%) | 0 (0.0%) | 1367 (67.1%) | <0.001 |
| **NOACs** | | 670 (26.4%) | 0 (0.0%) | 670 (32.9%) | <0.001 |
| **ACEI** | | 1051 (41.6%) | 206 (41.4%) | 845 (41.5%) | 0.963 |
| **ARBs** | | 589 (23.3%) | 84 (16.9%) | 505 (24.8%) | <0.001 |
| **Beta-blockers** | | 1746 (69.1%) | 320 (64.3%) | 1426 (70.0%) | 0.02 |

Continuous variables are presented as mean (standard deviation), while categorical variables are presented as number (percentage). CHA₂DS₂-VASc : Congestive Heart Failure, Hypertension, Age ≥75 (2 points), Diabetes Mellitus, Stroke/TIA/Thromboembolism (2 points), Vascular Disease, Age 65-74, Sex Category (Female); HAS-BLED : Hypertension, Abnormal renal/liver function, Stroke, Bleeding, Labile INR, Elderly, Drugs/Alcohol; LVEF : Left Ventricular Ejection Fraction; LVEDD : Left Ventricular End-Diastolic Diameter; VKA : Vitamin K Antagonists; NOAC : Non-Vitamin K Oral Anticoagulants; ACEI : Angiotensin-Converting Enzyme Inhibitor; ARB : Angiotensin II Receptor Blocker.

**Supplementary Table 6.** **Multivariable Logistic Regression Analysis of Clinical Outcomes.**

|  | **NACE**  **(OR 95% CI)** | **All-cause death**  **OR (95% CI)** | **MACE**  **OR (95% CI)** | **Major bleeding OR (95% CI)** | **Thromboembolic events**  **OR (95% CI)** | **Acute coronary syndrome**  **OR (95% CI)** |
| --- | --- | --- | --- | --- | --- | --- |
| **Age≥ 75 years** | 1.61(1.29-2.01) | 1.77(1.37- 2.29) | 1.32(1.03-1.68) | 0.99(0.66-1.51) | 0.78(0.48-1.25) | 1.27(0.86-1.89) |
| **Female sex** | 0.81(0.65-1.01) | 0.85(0.67-1.09) | 0.83(0.63-1.09) | 0.70(0.45-1.06) | 1.06(0.66-1.71) | 0.74(0.49-1.1) |
| **Hypertension** | 0.76(0.59-0.97) | 0.79(0.60-1.05) | 0.82(0.61-1.11) | 0.70(0.45-1.12) | 0.71(0.43-1.22) | 0.93(0.6-1.49) |
| **Diabetes** | 1.28(1.03-1.60) | 1.29(1.00-1.65) | 1.39(1.06-1.83) | 0.81(0.51-1.26) | 1.19(0.70-1.95) | 1.1(0.73-1.63) |
| **CKD** | 1.67(1.33-2.09) | 1.85(1.43-2.40) | 1.31(1.00-1.73) | 1.38(0.90-2.13) | 1.05(0.64-1.70) | 1.53(1.02-2.3) |
| **PAD** | 1.41(1.05-1.89) | 1.65(1.19-2.27) | 1.35(0.95-1.90) | 0.98(0.49-1.80) | 0.63(0.21-1.46) | 1.26(0.74-2.05) |
| **Dyslipidemia** | 0.84(0.67-1.04) | 0.75(0.59-0.96) | 0.75(0.57-0.98) | 1.19(0.78-1.81) | 0.45(0.27-0.75) | 1.01(0.68-1.49) |
| **COPD** | 1.20(0.87-1.66) | 1.36(0.96-1.91) | 0.81(0.52-1.22) | 0.59(0.24-1.23) | 0.24(0.04-0.78) | 0.94(0.5-1.65) |
| **Heart failure** | 1.39(1.11-1.73) | 1.50(1.16-1.94) | 1.41(1.07-1.87) | 1.06(0.69-1.63) | 0.97(0.59-1.58) | 0.95(0.64-1.41) |
| **Thromboembolic events** | 1.15(0.90-1.45) | 1.10(0.83-1.43) | 1.09(0.81-1.46) | 0.88(0.54-1.39) | 0.91(0.52-1.52) | 0.73(0.44-1.14) |
| **Hemorrhagic events** | 1.10(0.84-1.42) | 1.05(0.78-1.41) | 0.73(0.51-1.03) | 1.83(1.17-2.84) | 0.50(0.24-0.95) | 0.64(0.37-1.07) |
| **Antiplatelet use** | 1.20(0.95-1.52) | 0.93(0.71-1.21) | 1.33(1.00-1.77) | 0.61(0.38-0.98) | 0.41(0.22-0.73) | 1.84(1.21-2.81) |
| **Enrollment setting (APHRS + IRAF vs. EORP)** | 0.43(0.32-0.57) | 0.61(0.44-0.85) | 0.42(0.28-0.62) | 1.16(0.71-1.85) | 1.33(0.77-2.24) | 0.63(0.37-1.03) |
| **OAC *non-use*** | 2.15(1.63-2.82) | 2.23(1.65-3.01) | 1.92(1.38-2.64) | 2.38(1.42-3.92) | 2.53(1.34-4.61) | 2.09(1.34-3.25) |

The multivariable model assessing OAC use was adjusted for age ≥75 years, female sex, underweight, hypertension, diabetes mellitus (DM), chronic kidney disease (CKD), peripheral artery disease, dyslipidaemia, chronic obstructive pulmonary disease (COPD), heart failure, prior thromboembolic events, history of major bleeding, antiplatelet therapy, and enrolment setting (APHRS and IRAF vs. EORP). A separate multivariable model assessing the risk of adverse events was adjusted for these same covariates, plus baseline OAC use. NACE: Net adverse clinical events.

**Supplementary Table 7. Multivariable‐adjusted odds ratios (95% CI) for 1‑year clinical outcomes comparing OAC non‑users to VKA and NOAC users.**

|  | **NACE**  **(aOR 95% CI)** | **All-cause death**  **aOR (95% CI)** | **MACE**  **aOR (95% CI)** | **Major bleeding aOR (95% CI)** | **Thromboembolic events**  **aOR (95% CI)** | **Acute coronary syndrome**  **aOR (95% CI)** |
| --- | --- | --- | --- | --- | --- | --- |
| **OAC *non-use* vs. VKAs** | 2.05 (1.54-2.73) | 2.11 (0.48-3.03) | 1.92 (1.37–2.69) | 2.19 (1.29–3.72) | 2.70 (1.41–5.19) | 2.07(1.30–3.30) |
| **OAC *non-use* vs. NOACs** | 2.39 (1.71–3.34) | 2.54 (1.74–3.72) | 1.91 (1.27–2.86) | 2.83 (1.51–5.32) | 2.26 (1.12–4.56) | 2.16 (1.21–3.87) |
| **P for interaction** | 0.495 | 0.500 | 0.985 | 0.541 | 0.716 | 0.911 |
|  | **NACE**  **N (%)** | **All-cause death**  **N (%)** | **MACE**  **N (%)** | **Major bleeding**  **N (%)** | **Thromboembolic events**  **N (%)** | **Acute coronary syndrome**  **N (%)** |
| **OAC *non-users* (n=498)** | 167 (33.5%) | 116 (23.3%) | 96 (19.3%) | 31 (6.2%) | 21 (4.2%) | 51 (10.2%) |
| **VKA *users* (n=1367)** | 289 (21.1%) | 194 (14.2%) | 154 (11.3%) | 52 (3.8%) | 36 (2.6%) | 61 (4.5%) |
| **NOAC *users* (n=670)** | 111 (16.6%) | 70 (10.4%) | 66 (9.9%) | 21 (3.1%) | 27 (4.0%) | 24 (3.6%) |

The multivariable model was adjusted for age ≥75 years, female sex, underweight, hypertension, diabetes mellitus (DM), chronic kidney disease (CKD), peripheral artery disease, dyslipidaemia, chronic obstructive pulmonary disease (COPD), heart failure, prior thromboembolic events, history of major bleeding, antiplatelet therapy, and enrolment setting (APHRS and IRAF vs. EORP). aOR: adjusted odds ratio. NACE: net adverse clinical events.

**Supplementary Table 8. Association between oral anticoagulant (OAC) non-use and composite outcome stratified by CHA₂DS₂-VASc and HAS-BLED categories**

| **Risk score** | **Adjusted OR (95% CI)** |
| --- | --- |
| **High thromboembolic risk**  **(CHA₂DS₂-VASc score of 3–5)** | 2.03 (1.43–2.86) |
| **Very high thromboembolic risk**  **(CHA₂DS₂-VASc score of 6-9)** | 3.30 (1.97–5.53) |
| **High bleeding risk (HAS-BLED score of 3-4)** | 2.22 (1.65–2.98) |
| **Very high bleeding risk (HAS-BLED score of 5–9)** | 5.62 (1.60–19.78) |

Adjusted odds ratios (ORs) are derived from multivariable logistic regression models fitted separately within each risk stratum, with OAC non-use as the main independent variable and OAC use as the reference. Models were adjusted for age ≥75 years, sex, hypertension, diabetes, chronic kidney disease (eGFR <60 mL/min by MDRD), peripheral arterial disease, dyslipidaemia, chronic obstructive pulmonary disease, heart failure, prior thromboembolic events, prior haemorrhagic events, antiplatelet use, and enrolment setting (APHRS + IRAF vs EORP).

**Supplementary Table 9. Time-to-event sensitivity analyses using Cox proportional hazards models.**

| **Predictor** | **NACE**  **aHR (95% CI)** | **Thromboembolic events**  **aHR (95% CI)** | **Major bleeding**  **aHR (95% CI)** |
| --- | --- | --- | --- |
| **OAC non-use** | 1.46 (1.09–1.96) | 1.41 (0.56–3.56) | 1.56 (0.81–3.02) |
| **Age ≥75 years** | 1.65 (1.28–2.13) | 1.13 (0.55–2.31) | 1.33 (0.77–2.30) |
| **Female sex** | 0.80 (0.63–1.01) | 1.24 (0.61–2.52) | 0.47 (0.26–0.85) |
| **Hypertension** | 0.88 (0.67–1.15) | 0.86 (0.39–1.88) | 0.87 (0.48–1.59) |
| **Diabetes** | 1.28 (1.00–1.63) | 1.39 (0.67–2.89) | 0.94 (0.53–1.66) |
| **CKD (eGFR <60 mL/min)** | 1.72 (1.34–2.22) | 1.36 (0.66–2.83) | 1.42 (0.82–2.46) |
| **Peripheral arterial disease** | 1.33 (0.98–1.80) | 0.66 (0.20–2.23) | 1.14 (0.55–2.40) |
| **Dyslipidaemia** | 0.86 (0.68–1.09) | 0.42 (0.19–0.90) | 1.17 (0.68–2.01) |
| **COPD** | 1.18 (0.84–1.65) | 0.24 (0.03–1.75) | 0.52 (0.18–1.45) |
| **Heart failure** | 1.35 (1.05–1.73) | 0.88 (0.43–1.81) | 1.17 (0.68–2.03) |
| **History of thromboembolism** | 1.30 (1.01–1.68) | 1.67 (0.81–3.45) | 1.17 (0.66–2.09) |
| **History of major bleeding** | 1.32 (1.00–1.73) | 0.82 (0.31–2.16) | 2.64 (1.52–4.57) |
| **Antiplatelet therapy** | 1.23 (0.95–1.59) | 0.94 (0.42–2.08) | 1.03 (0.57–1.85) |
| **Asian ethnicity (APHRS/IRAF vs EORP)** | 0.62 (0.45–0.85) | 0.25 (0.07–0.83) | 0.52 (0.26–1.05) |

Hazard ratios (HR) and 95% confidence intervals (CI) for the association between baseline covariates and: (1) the composite outcome of thromboembolic events, acute coronary syndrome, cardiovascular events, major bleeding, or death; (2) thromboembolic events, censoring at the first occurrence of major bleeding or death; and (3) major bleeding, censoring at the first thromboembolic event, acute coronary syndrome, cardiovascular event, or death. All endpoints were assessed within 1 year of baseline. All models were adjusted for the covariates listed in the table. HR > 1 indicates a higher hazard of the specified outcome.

**Supplementary Table 10. Inverse probability of treatment weighting (IPTW)–weighted odds of outcomes according to oral anticoagulant (OAC) use.**

| **Outcome** | **Comparison** | **OR (95% CI)** |
| --- | --- | --- |
| **Composite outcome (NACE)** | OAC *use* | Ref |
|  | OAC *non-use* | 2.92 (2.27–3.76) |
| **Major bleeding** | OAC *use* | Ref |
|  | OAC *non-use* | 2.69 (1.70–4.17) |
| **Cardiovascular events (MACE)** | OAC *use* | Ref |
|  | OAC *non-use* | 2.97 (2.23–3.94) |
| **All-cause death** | OAC *use* | Ref |
|  | OAC *non-use* | 2.69 (2.05–3.53) |
| **Acute coronary syndrome (ACS)** | OAC *use* | Ref |
|  | OAC *non-use* | 2.84 (1.88–4.21) |
| **Thromboembolic events (TE)** | OAC *use* | Ref |
|  | OAC *non-use* | 3.55 (2.22–5.60) |

Odds ratios (OR) and 95% confidence intervals (CI) for the association between baseline oral anticoagulant (OAC) non-use and 1-year outcomes in the inverse probability of treatment weighting (IPTW) pseudo-population. Propensity scores for OAC use were estimated using a logistic regression model including age, sex, body mass index, hypertension, diabetes, chronic kidney disease (eGFR <60 mL/min), peripheral arterial disease, dyslipidaemia, COPD, heart failure, history of thromboembolism, history of major bleeding, antiplatelet therapy, and registry (Asian vs non-Asian setting). Stabilized IPTW weights were derived and truncated at the 1st and 99th percentiles. Separate weighted logistic regression models were then fitted for each endpoint, with exposure coded as no OAC versus OAC. NACE: net adverse clinical events; MACE: major adverse cardiovascular events; ACS: acute coronary syndrome; TE: thromboembolic events; eGFR: estimated glomerular filtration rate; COPD: chronic obstructive pulmonary disease.

**Supplementry Table 11. Fine–Gray competing risks models for 1-year thromboembolic events and major bleeding.**

| **Event of interest** | **Thromboembolic events**  **SHR (95% CI)** | **Major bleeding**  **SHR (95% CI)** |
| --- | --- | --- |
| **OAC non-use** | 1.18 (0.47–2.94) | 1.45 (0.74–2.83) |
| **Age ≥75 years** | 1.07 (0.51–2.22) | 1.47 (0.84–2.57) |
| **Female sex** | 1.19 (0.56–2.51) | 0.50 (0.28–0.91) |
| **Hypertension** | 0.80 (0.36–1.77) | 0.83 (0.46–1.53) |
| **Diabetes mellitus** | 1.45 (0.68–3.09) | 0.89 (0.51–1.56) |
| **CKD (eGFR <60 mL/min)** | 1.27 (0.62–2.61) | 1.28 (0.76–2.17) |
| **Peripheral artery disease** | 0.67 (0.19–2.30) | 1.01 (0.45–2.28) |
| **Dyslipidaemia** | 0.45 (0.20–0.98) | 1.24 (0.73–2.12) |
| **COPD** | 0.24 (0.03–1.85) | 0.51 (0.18–1.49) |
| **Heart failure** | 0.81 (0.42–1.59) | 1.19 (0.70–2.01) |
| **Prior thromboembolism** | 1.70 (0.81–3.55) | 1.13 (0.61–2.08) |
| **Prior haemorrhagic events** | 0.64 (0.23–1.81) | 2.61 (1.53–4.45) |
| **Antiplatelet therapy** | 0.90 (0.40–2.01) | 1.04 (0.56–1.92) |
| **Enrollment in Asian setting** | 0.26 (0.08–0.83) | 0.48 (0.23–1.01) |

Data are subdistribution hazard ratios (SHR) from Fine–Gray competing risk models with 95% confidence intervals (CI). ACS = acute coronary syndrome; CKD = chronic kidney disease; COPD = chronic obstructive pulmonary disease; eGFR = estimated glomerular filtration rate; OAC = oral anticoagulant. Competing events for Thromboembolic events were major bleeding or death. Competing events for major bleeding were thromboembolism, ACS, other cardiovascular events, or death.

**Supplementary Figure 1. Propensity score distributions before and after inverse probability of treatment weighting (IPTW).** Panel A shows the unweighted kernel density distributions of the estimated propensity score for oral anticoagulant (OAC) use versus non-use (No OAC). Panel B shows the corresponding distributions after IPTW with weight truncation. The x-axis denotes the propensity score, and the y-axis denotes probability density.

**Supplementary Figure 2. Subgroup analyses for the risk of all-cause death among OAC *users* versus OAC *non-users.*** HTN: hypertension; COPD: chronic obstructive pulmonary disease; CKD: chronic kidney disease; HF: heart failure; PAD: peripheral artery disease.

**Supplementary Figure 3. Cumulative incidence functions by OAC status for 1-year thromboembolic events with major bleeding or death as competing events.** Solid lines represent thromboembolic events, and dashed lines represent competing events; blue lines indicate OAC use, and red lines indicate OAC non-use. Among 2,318 patients, 31 thromboembolic events, 243 competing events, and 2,044 censored observations were observed.

**Supplementary Figure 4. Cumulative incidence functions by OAC status for 1-year major bleeding and competing events (thromboembolism, acute coronary syndrome, other cardiovascular events, or death).** Solid lines represent major bleeding, and dashed lines represent competing events; blue lines indicate OAC use, and red lines indicate OAC non-use. In 2,284 patients, 55 major bleeding events, 234 competing events, and 1,995 censored observations occurred.

**References**:

1. Boriani G, Proietti M, Laroche C, Fauchier L, Marin F, Nabauer M, et al. Association between antithrombotic treatment and outcomes at 1-year follow-up in patients with atrial fibrillation: the EORP-AF General Long-Term Registry. EP Europace. 2019;21(7):1013-22.

2. Tse H-F, Teo W-S, Siu C-W, Chao T-F, Park H-W, Shimizu W, et al. Prognosis and treatment of atrial fibrillation in Asian cities: 1-year review of the Asia-Pacific Heart Rhythm Society Atrial Fibrillation Registry. EP Europace. 2022;24(12):1889-98.

3. Haghjoo M, Askarinejad A, Heidarali M, Bakhshandeh H, Fazelifar A, Emkanjoo Z, et al. Implementation of an atrial fibrillation better care (ABC) pathway management strategy: Findings from the Iranian registry of atrial fibrillation. IJC Heart & Vasculature. 2024;53:101461.
