# Supplementary figures and images for: Real-world outcomes of oral anticoagulation in patients with atrial fibrillation at high risk of both bleeding and stroke: observational evidence from three international registries from middle East, Europe and Asia-Pacific
Source: J Thromb Thrombolysis. 2025 Dec 22;59(3):574–86. doi: 10.1007/s11239-025-03228-6 (PMC13246897; doi:10.1007/s11239-025-03228-6)

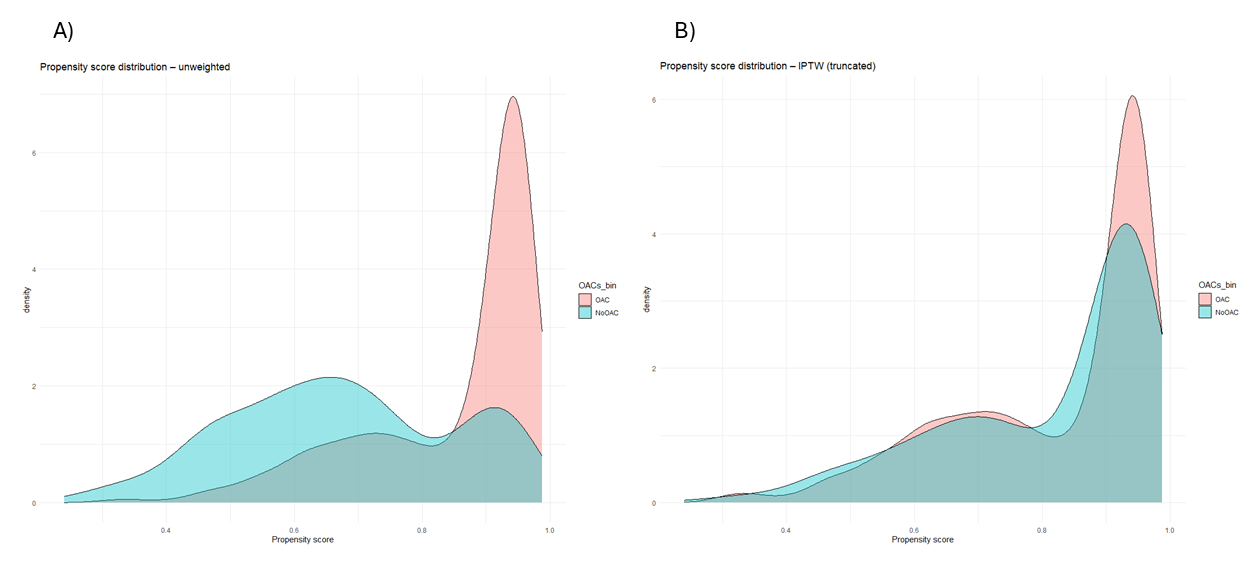

Supplement: Supplementary file 2 — Supplementary Figure 1. Propensity score distributions before and after inverse probability of treatment weighting (IPTW). [file 11239_2025_3228_MOESM2_ESM.png]

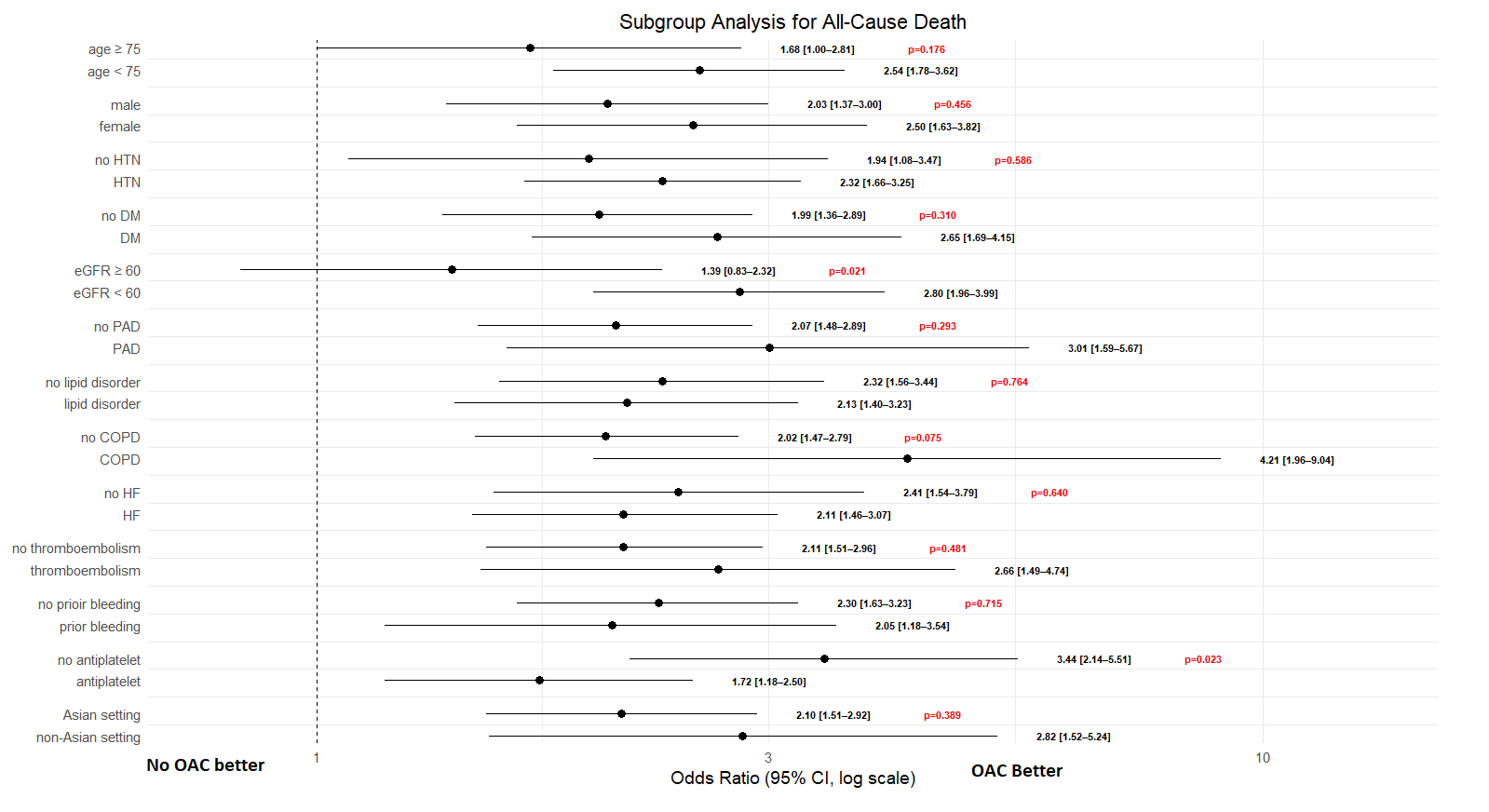

Supplement: Supplementary file 3 — Supplementary Material 3Supplementary Figure 2. Subgroup analyses for the risk of all-cause death among OAC users versus OAC non-users [file 11239_2025_3228_MOESM3_ESM.png]

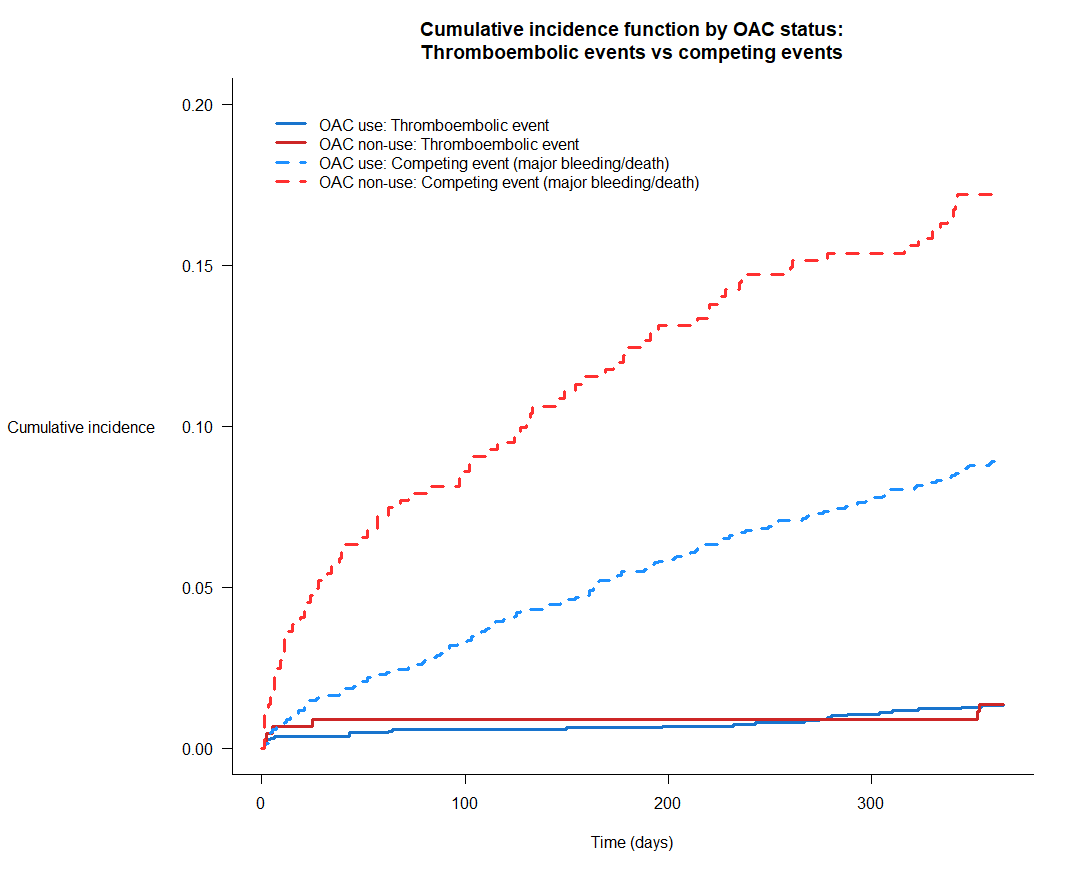

Supplement: Supplementary file 4 — Supplementary Figure 3. Cumulative incidence functions by OAC status for 1-year thromboembolic events with major bleeding or death as competing events. [file 11239_2025_3228_MOESM4_ESM.png]

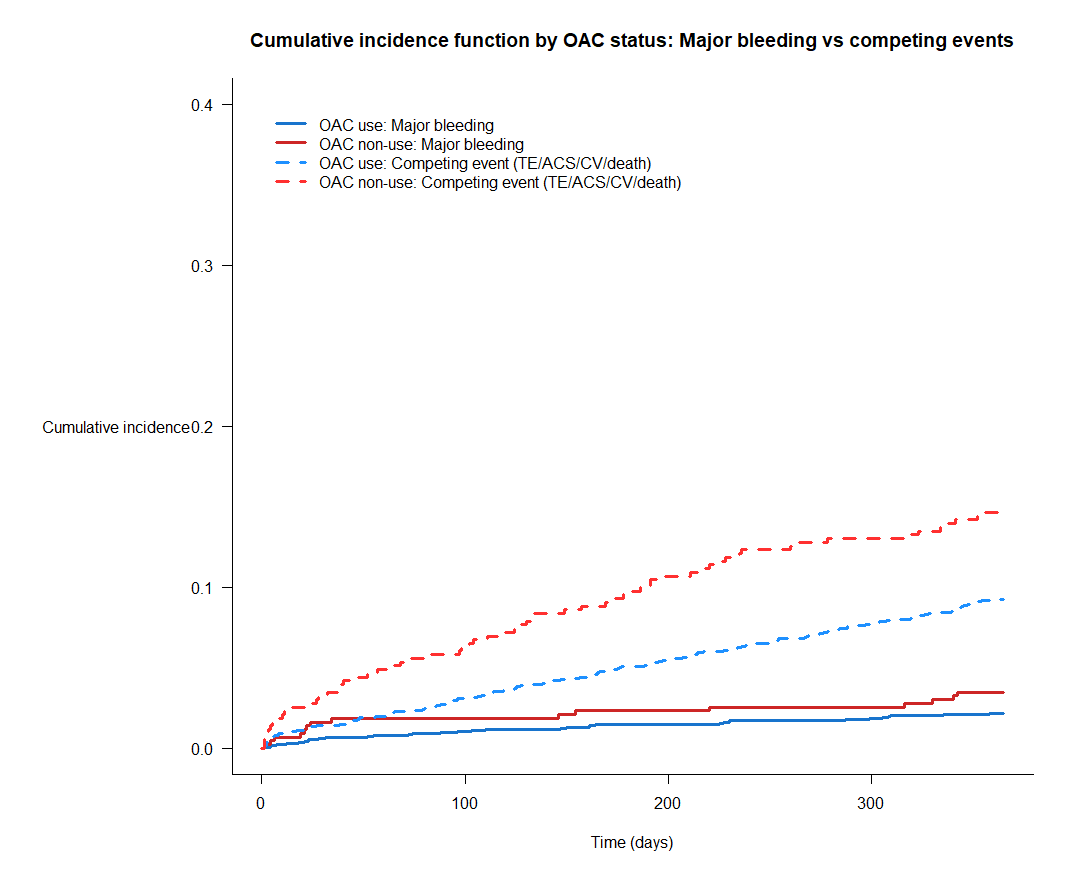

Supplement: Supplementary file 5 — Supplementary Figure 4. Cumulative incidence functions by OAC status for 1-year major bleeding and competing events (thromboembolism, acute coronary syndrome, other cardiovascular events, or death). [file 11239_2025_3228_MOESM5_ESM.png]
